# Supplementary material for: The WAC-downWAC domain in the yeast ISW2 nucleosome remodeling complex forms a structural module essential for ISW2 function but not cell viability
Source: Epigenetics Chromatin. 2025 May 21;18:30. doi: 10.1186/s13072-025-00593-7 (PMC12093815; doi:10.1186/s13072-025-00593-7)
Supplement: Supplementary file 1 — Supplementary Material 1. [file 13072_2025_593_MOESM1_ESM.docx]

**Table S1: Quantification of nucleosome shift in mutant strains relative to WT based on composite plots. Related to Fig. 4A.** Positive values indicate a downstream shift and negative values indicate an upstream shift relative to the corresponding WT nucleosome in the composite plot. “NA” indicates cases when a clear nucleosome peak is not identified due to extremely fuzzy nucleosomes in the composite plots.

|  | **Nuc +1** | **Nuc +2** | **Nuc +3** | **Nuc +4** | **Nuc +5** | **Nuc +6** |
| --- | --- | --- | --- | --- | --- | --- |
| *isw2Δ* | +24 | +23 | +17 | +12 | +10 | +8 |
| *itc1Δ* | +24 | +22 | +18 | +13 | +9 | +6 |
| *itc1ΔN* | +24 | +23 | +18 | +11 | +10 | +6 |
| *isw2Δ itc1ΔN* | +25 | +24 | +16 | +12 | +8 | +5 |
| *isw1Δ chd1Δ* | -4 | +2 | +5 | +15 | NA | NA |
| *isw1Δ chd1Δ*  *isw2Δ* | +25 | +23 | +21 | +25 | NA | NA |
| *isw1Δ chd1Δ*  *itc1ΔN* | +23 | +22 | +22 | +25 | NA | NA |

**Table S2: List of genes showing +1 nucleosome shift by at least 10 bp in indicated yeast strains compared to WT.**

| *isw2Δ* | *itc1Δ* | *itc1^ΔN^* | *isw2Δ itc1^ΔN^* | *isw1Δ chd1Δ* | *isw1Δ chd1Δ isw2Δ* | *isw1Δ chd1Δ itc1^ΔN^* |
| --- | --- | --- | --- | --- | --- | --- |
| YAL018C | YAL018C | YAL018C | YAL002W | YAL042W | YAL042W | YAL042W |
| YAL043C | YAL043C | YAL043C | YAL018C | YBL004W | YAL043C | YAL043C |
| YAR015W | YAL060W | YAR018C | YAL043C | YBR085W | YAL054C | YAR015W |
| YAR018C | YAR018C | YBL023C | YAR015W | YBR182C | YAR015W | YAR018C |
| YBL023C | YBL023C | YBL034C | YAR018C | YBR199W | YAR018C | YBL042C |
| YBL034C | YBL034C | YBL042C | YBL023C | YBR202W | YAR018C | YBL106C |
| YBL042C | YBL042C | YBL085W | YBL034C | YBR297W | YBL023C | YBR002C |
| YBL085W | YBL085W | YBR021W | YBL042C | YCL051W | YBL042C | YBR038W |
| YBR021W | YBR021W | YBR038W | YBL085W | YCL064C | YBR021W | YBR039W |
| YBR038W | YBR038W | YBR055C | YBR021W | YCR088W | YBR021W | YBR046C |
| YBR055C | YBR055C | YBR085W | YBR038W | YDL101C | YBR021W | YBR085W |
| YBR150C | YBR085W | YBR150C | YBR055C | YDL120W | YBR026C | YBR095C |
| YBR160W | YBR150C | YBR160W | YBR085W | YDR063W | YBR038W | YBR102C |
| YBR177C | YBR160W | YBR177C | YBR150C | YDR196C | YBR085W | YBR150C |
| YBR182C | YBR177C | YBR182C | YBR160W | YDR393W | YBR085W | YBR172C |
| YBR202W | YBR182C | YBR202W | YBR182C | YDR456W | YBR092C | YBR182C |
| YBR210W | YBR202W | YBR210W | YBR202W | YEL072W | YBR150C | YBR182C |
| YBR271W | YBR210W | YBR271W | YBR210W | YER074W-A | YBR162W-A | YBR202W |
| YBR291C | YBR271W | YBR291C | YBR271W | YER146W | YBR182C | YBR210W |
| YBR296C | YBR291C | YBR296C | YBR291C | YER149C | YBR182C | YBR213W |
| YBR297W | YBR296C | YBR297W | YBR296C | YFR011C | YBR202W | YBR291C |
| YBR298C | YBR297W | YBR298C | YBR297W | YGL009C | YBR202W | YBR296C |
| YCL024W | YBR298C | YCL024W | YBR298C | YGL196W | YBR210W | YBR297W |
| YCR031C | YCL024W | YCR031C | YCL024W | YGR199W | YBR218C | YCL047C |
| YCR045C | YCR045C | YCR045C | YCR031C | YHR014W | YBR233W | YCL059C |
| YCR053W | YCR053W | YCR053W | YCR045C | YHR096C | YBR291C | YCR044C |
| YDL003W | YDL003W | YDL003W | YCR053W | YHR133C | YBR291C | YCR045C |
| YDL101C | YDL101C | YDL101C | YDL003W | YHR157W | YBR296C | YCR045C |
| YDL144C | YDL144C | YDL144C | YDL101C | YHR168W | YBR297W | YCR052W |
| YDL154W | YDL154W | YDL154W | YDL144C | YIL013C | YBR297W | YCR053W |
| YDL171C | YDL171C | YDL171C | YDL154W | YIL131C | YBR298C | YDL003W |
| YDL194W | YDL194W | YDL194W | YDL171C | YJL002C | YCL027W | YDL019C |
| YDR019C | YDR019C | YDR019C | YDL194W | YJR078W | YCL045C | YDL040C |
| YDR043C | YDR043C | YDR043C | YDR019C | YJR110W | YCR045C | YDL101C |
| YDR059C | YDR059C | YDR059C | YDR043C | YKL015W | YCR045C | YDL110C |
| YDR146C | YDR146C | YDR146C | YDR059C | YKL033W-A | YCR053W | YDL111C |
| YDR213W | YDR213W | YDR213W | YDR146C | YKL093W | YDL003W | YDL131W |
| YDR247W | YDR247W | YDR247W | YDR213W | YKL185W | YDL003W | YDL144C |
| YDR256C | YDR256C | YDR256C | YDR247W | YLR058C | YDL097C | YDL154W |
| YDR263C | YDR263C | YDR263C | YDR256C | YLR081W | YDL101C | YDR063W |
| YDR272W | YDR272W | YDR272W | YDR272W | YLR312C | YDL101C | YDR096W |
| YDR277C | YDR277C | YDR277C | YDR277C | YLR409C | YDL144C | YDR146C |
| YDR311W | YDR311W | YDR311W | YDR311W | YML042W | YDL154W | YDR169C |
| YDR317W | YDR317W | YDR317W | YDR317W | YMR018W | YDL154W | YDR175C |
| YDR374C | YDR374C | YDR374C | YDR374C | YMR189W | YDL182W | YDR213W |
| YDR375C | YDR375C | YDR375C | YDR375C | YNL041C | YDL226C | YDR225W |
| YDR393W | YDR393W | YDR393W | YDR393W | YNR015W | YDR007W | YDR229W |
| YDR403W | YDR403W | YDR403W | YDR403W | YOR036W | YDR043C | YDR256C |
| YDR451C | YDR451C | YDR451C | YDR451C | YOR066W | YDR142C | YDR263C |
| YDR507C | YDR507C | YDR507C | YDR507C | YOR229W | YDR146C | YDR272W |
| YDR524C | YDR524C | YDR524C | YDR524C | YPL011C | YDR146C | YDR277C |
| YDR528W | YDR528W | YDR528W | YDR528W | YPL088W | YDR204W | YDR294C |
| YDR532C | YDR532C | YDR532C | YDR532C | YPL104W | YDR238C | YDR295C |
| YEL017C-A | YEL017C-A | YEL017C-A | YEL017C-A | YPL264C | YDR247W | YDR311W |
| YEL030W | YEL030W | YEL030W | YEL030W |  | YDR256C | YDR317W |
| YEL039C | YEL039C | YEL039C | YEL039C |  | YDR263C | YDR317W |
| YEL042W | YEL042W | YEL042W | YEL042W |  | YDR263C | YDR341C |
| YEL072W | YEL072W | YEL072W | YEL072W |  | YDR272W | YDR347W |
| YER017C | YER017C | YER017C | YER017C |  | YDR277C | YDR349C |
| YER044C-A | YER044C-A | YER044C-A | YER044C-A |  | YDR306C | YDR374C |
| YER070W | YER070W | YER062C | YER070W |  | YDR311W | YDR389W |
| YER091C | YER091C | YER070W | YER091C |  | YDR317W | YDR393W |
| YER095W | YER095W | YER091C | YER095W |  | YDR357C | YDR395W |
| YER096W | YER096W | YER095W | YER096W |  | YDR358W | YDR403W |
| YER123W | YER123W | YER096W | YER123W |  | YDR374C | YDR408C |
| YER146W | YER146W | YER123W | YER146W |  | YDR375C | YDR424C |
| YER155C | YER155C | YER146W | YER155C |  | YDR393W | YDR451C |
| YFL052W | YFL052W | YER155C | YFL052W |  | YDR403W | YDR483W |
| YFL054C | YFL054C | YFL052W | YFL054C |  | YDR451C | YDR507C |
| YFR011C | YFR011C | YFL054C | YFR011C |  | YDR458C | YDR531W |
| YFR015C | YFR015C | YFR011C | YFR015C |  | YDR472W | YDR532C |
| YFR016C | YFR016C | YFR015C | YFR016C |  | YDR507C | YEL030W |
| YFR028C | YFR028C | YFR016C | YFR028C |  | YDR528W | YEL039C |
| YGL021W | YGL021W | YFR028C | YGL021W |  | YDR532C | YEL039C |
| YGL116W | YGL116W | YGL021W | YGL116W |  | YDR540C | YEL067C |
| YGL117W | YGL117W | YGL116W | YGL117W |  | YEL030W | YEL072W |
| YGL254W | YGL254W | YGL117W | YGL254W |  | YEL039C | YER009W |
| YGR087C | YGR087C | YGL254W | YGR087C |  | YEL039C | YER015W |
| YGR092W | YGR092W | YGR087C | YGR092W |  | YEL065W | YER044C-A |
| YGR129W | YGR129W | YGR092W | YGR129W |  | YEL072W | YER051W |
| YGR161C | YGR161C | YGR129W | YGR161C |  | YEL072W | YER068W |
| YGR177C | YGR177C | YGR161C | YGR177C |  | YER017C | YER070W |
| YGR199W | YGR199W | YGR177C | YGR199W |  | YER036C | YER091C |
| YGR221C | YGR221C | YGR199W | YGR221C |  | YER044C-A | YER095W |
| YGR240C | YGR240C | YGR221C | YGR240C |  | YER070W | YER096W |
| YGR248W | YGR248W | YGR240C | YGR248W |  | YER070W | YER126C |
| YGR281W | YGR281W | YGR248W | YGR281W |  | YER078C | YER128W |
| YGR289C | YGR289C | YGR281W | YGR289C |  | YER091C | YER140W |
| YHL026C | YHL026C | YGR289C | YHL026C |  | YER095W | YER146W |
| YHR014W | YHR014W | YHL026C | YHR014W |  | YER095W | YER151C |
| YHR032W | YHR032W | YHR014W | YHR032W |  | YER096W | YFL026W |
| YHR043C | YHR043C | YHR032W | YHR043C |  | YER096W | YFL047W |
| YHR048W | YHR048W | YHR043C | YHR048W |  | YER146W | YFL054C |
| YHR061C | YHR061C | YHR048W | YHR061C |  | YER146W | YFR011C |
| YHR096C | YHR096C | YHR061C | YHR096C |  | YER176W | YFR028C |
| YHR122W | YHR122W | YHR096C | YHR122W |  | YFL014W | YFR035C |
| YHR124W | YHR124W | YHR122W | YHR124W |  | YFL048C | YGL016W |
| YHR137W | YHR137W | YHR124W | YHR137W |  | YFL054C | YGL021W |
| YHR149C | YHR149C | YHR137W | YHR149C |  | YFL054C | YGL078C |
| YHR153C | YHR153C | YHR149C | YHR153C |  | YFR011C | YGL087C |
| YHR154W | YHR154W | YHR153C | YHR154W |  | YFR016C | YGL116W |
| YHR156C | YHR156C | YHR154W | YHR156C |  | YGL021W | YGL117W |
| YHR157W | YHR157W | YHR156C | YHR157W |  | YGL021W | YGL135W |
| YHR160C | YHR160C | YHR157W | YHR160C |  | YGL037C | YGL211W |
| YHR210C | YHR210C | YHR160C | YHR210C |  | YGL039W | YGL234W |
| YIL013C | YIL013C | YHR210C | YIL013C |  | YGL106W | YGL254W |
| YIL020C | YIL020C | YIL013C | YIL020C |  | YGL116W | YGR083C |
| YIL057C | YIL057C | YIL020C | YIL057C |  | YGL117W | YGR087C |
| YIL072W | YIL072W | YIL057C | YIL072W |  | YGL221C | YGR087C |
| YIL099W | YIL099W | YIL072W | YIL099W |  | YGL232W | YGR113W |
| YIL117C | YIL117C | YIL099W | YIL117C |  | YGL254W | YGR177C |
| YIL131C | YIL131C | YIL117C | YIL131C |  | YGR065C | YGR199W |
| YIL138C | YIL138C | YIL131C | YIL138C |  | YGR087C | YGR221C |
| YIL158W | YIL158W | YIL138C | YIL160C |  | YGR087C | YGR223C |
| YIL160C | YIL160C | YIL158W | YIR034C |  | YGR108W | YGR247W |
| YIR034C | YIR034C | YIL160C | YJL051W |  | YGR129W | YGR248W |
| YJL051W | YJL051W | YIR034C | YJL061W |  | YGR177C | YGR257C |
| YJL061W | YJL061W | YJL051W | YJL088W |  | YGR199W | YHR014W |
| YJL088W | YJL088W | YJL061W | YJL151C |  | YGR213C | YHR016C |
| YJL153C | YJL153C | YJL088W | YJL153C |  | YGR221C | YHR017W |
| YJL159W | YJL159W | YJL153C | YJL159W |  | YGR221C | YHR018C |
| YJL177W | YJL177W | YJL159W | YJL177W |  | YGR243W | YHR032W |
| YJL194W | YJL194W | YJL177W | YJL194W |  | YGR248W | YHR043C |
| YJL214W | YJL214W | YJL194W | YJL214W |  | YGR274C | YHR061C |
| YJL216C | YJL216C | YJL214W | YJL216C |  | YGR289C | YHR094C |
| YJR015W | YJR015W | YJL216C | YJR015W |  | YGR289C | YHR096C |
| YJR030C | YJR030C | YJR015W | YJR030C |  | YHR014W | YHR096C |
| YJR036C | YJR036C | YJR030C | YJR036C |  | YHR032W | YHR119W |
| YJR061W | YJR061W | YJR036C | YJR061W |  | YHR043C | YHR124W |
| YJR078W | YJR078W | YJR061W | YJR078W |  | YHR044C | YHR137W |
| YJR095W | YJR092W | YJR078W | YJR092W |  | YHR048W | YHR149C |
| YJR110W | YJR095W | YJR092W | YJR095W |  | YHR061C | YHR157W |
| YJR150C | YJR110W | YJR095W | YJR110W |  | YHR096C | YHR158C |
| YJR151C | YJR150C | YJR110W | YJR150C |  | YHR096C | YHR186C |
| YKL015W | YJR151C | YJR150C | YJR151C |  | YHR122W | YHR210C |
| YKL045W | YKL009W | YJR151C | YKL015W |  | YHR124W | YIL013C |
| YKL047W | YKL015W | YKL015W | YKL045W |  | YHR137W | YIL072W |
| YKL093W | YKL045W | YKL045W | YKL047W |  | YHR137W | YIL099W |
| YKL096W | YKL047W | YKL047W | YKL093W |  | YHR137W | YIL113W |
| YKL143W | YKL093W | YKL093W | YKL096W |  | YHR149C | YIL117C |
| YKL164C | YKL096W | YKL096W | YKL143W |  | YHR149C | YIL119C |
| YKL185W | YKL143W | YKL143W | YKL164C |  | YHR149C | YIL127C |
| YKL208W | YKL164C | YKL164C | YKL185W |  | YHR153C | YIL131C |
| YKR009C | YKL185W | YKL185W | YKL208W |  | YHR156C | YIL138C |
| YKR097W | YKL208W | YKL208W | YKR009C |  | YHR157W | YIL158W |
| YLL021W | YKR009C | YKR009C | YKR097W |  | YHR157W | YIL160C |
| YLL046C | YKR097W | YKR097W | YLL021W |  | YHR160C | YIR001C |
| YLL056C | YLL021W | YLL021W | YLL046C |  | YHR210C | YIR011C |
| YLR054C | YLL046C | YLL046C | YLL056C |  | YHR210C | YIR034C |
| YLR058C | YLL056C | YLL056C | YLR054C |  | YIL013C | YJL023C |
| YLR081W | YLR054C | YLR054C | YLR058C |  | YIL013C | YJL031C |
| YLR084C | YLR058C | YLR058C | YLR081W |  | YIL020C | YJL042W |
| YLR179C | YLR081W | YLR081W | YLR084C |  | YIL036W | YJL051W |
| YLR183C | YLR084C | YLR084C | YLR179C |  | YIL057C | YJL088W |
| YLR249W | YLR179C | YLR168C | YLR183C |  | YIL072W | YJL127C |
| YLR254C | YLR183C | YLR179C | YLR249W |  | YIL072W | YJL128C |
| YLR263W | YLR249W | YLR183C | YLR254C |  | YIL073C | YJL153C |
| YLR267W | YLR254C | YLR249W | YLR263W |  | YIL099W | YJL159W |
| YLR312C | YLR263W | YLR254C | YLR267W |  | YIL117C | YJL194W |
| YLR313C | YLR267W | YLR263W | YLR312C |  | YIL131C | YJL214W |
| YLR407W | YLR312C | YLR267W | YLR313C |  | YIL138C | YJL216C |
| YLR441C | YLR313C | YLR312C | YLR407W |  | YIL158W | YJR030C |
| YML042W | YLR407W | YLR313C | YLR441C |  | YIL158W | YJR030C |
| YML059C | YLR441C | YLR407W | YML042W |  | YIL158W | YJR061W |
| YML119W | YML042W | YLR441C | YML059C |  | YIL160C | YJR075W |
| YMR002W | YML059C | YML042W | YML119W |  | YIL166C | YJR078W |
| YMR006C | YML119W | YML059C | YMR002W |  | YIR034C | YJR082C |
| YMR018W | YMR002W | YML119W | YMR006C |  | YIR034C | YJR088C |
| YMR032W | YMR006C | YMR002W | YMR018W |  | YJL045W | YJR092W |
| YMR055C | YMR018W | YMR006C | YMR032W |  | YJL051W | YJR095W |
| YMR076C | YMR032W | YMR018W | YMR055C |  | YJL051W | YJR124C |
| YMR144W | YMR055C | YMR032W | YMR076C |  | YJL088W | YJR150C |
| YMR189W | YMR076C | YMR055C | YMR144W |  | YJL088W | YJR151C |
| YMR240C | YMR144W | YMR076C | YMR189W |  | YJL093C | YKL003C |
| YMR278W | YMR189W | YMR144W | YMR240C |  | YJL145W | YKL015W |
| YMR280C | YMR240C | YMR177W | YMR266W |  | YJL153C | YKL047W |
| YNL012W | YMR278W | YMR189W | YMR278W |  | YJL159W | YKL052C |
| YNL014W | YMR280C | YMR240C | YMR280C |  | YJL159W | YKL093W |
| YNL037C | YNL012W | YMR278W | YNL012W |  | YJL162C | YKL096W |
| YNL058C | YNL014W | YMR280C | YNL014W |  | YJL194W | YKL129C |
| YNL112W | YNL037C | YNL012W | YNL037C |  | YJL194W | YKL130C |
| YNL183C | YNL041C | YNL014W | YNL041C |  | YJL214W | YKL143W |
| YNL196C | YNL058C | YNL037C | YNL058C |  | YJL216C | YKL173W |
| YNL208W | YNL112W | YNL041C | YNL112W |  | YJL216C | YKL185W |
| YNL234W | YNL183C | YNL058C | YNL183C |  | YJR036C | YKL186C |
| YNL270C | YNL196C | YNL112W | YNL196C |  | YJR036C | YKL208W |
| YNL279W | YNL208W | YNL183C | YNL208W |  | YJR036C | YKL218C |
| YNL306W | YNL234W | YNL196C | YNL234W |  | YJR039W | YKR005C |
| YNR002C | YNL259C | YNL208W | YNL270C |  | YJR061W | YKR009C |
| YNR015W | YNL270C | YNL234W | YNL306W |  | YJR078W | YKR017C |
| YNR061C | YNL279W | YNL270C | YNR002C |  | YJR092W | YKR037C |
| YOL007C | YNL306W | YNL279W | YNR015W |  | YJR095W | YKR051W |
| YOL014W | YNR002C | YNL306W | YNR061C |  | YJR104C | YKR084C |
| YOL017W | YNR015W | YNR002C | YOL007C |  | YJR123W | YKR097W |
| YOL066C | YNR061C | YNR015W | YOL014W |  | YJR150C | YLL003W |
| YOL084W | YOL007C | YNR061C | YOL017W |  | YJR151C | YLL021W |
| YOL091W | YOL014W | YOL007C | YOL034W |  | YJR151C | YLL048C |
| YOL104C | YOL017W | YOL014W | YOL066C |  | YKL015W | YLL056C |
| YOL151W | YOL034W | YOL017W | YOL084W |  | YKL015W | YLR018C |
| YOR009W | YOL066C | YOL030W | YOL091W |  | YKL047W | YLR028C |
| YOR019W | YOL084W | YOL034W | YOL104C |  | YKL093W | YLR054C |
| YOR058C | YOL091W | YOL066C | YOL151W |  | YKL093W | YLR067C |
| YOR062C | YOL104C | YOL084W | YOR009W |  | YKL096W | YLR081W |
| YOR066W | YOL151W | YOL091W | YOR019W |  | YKL096W | YLR084C |
| YOR083W | YOR009W | YOL100W | YOR058C |  | YKL143W | YLR100W |
| YOR100C | YOR019W | YOL104C | YOR062C |  | YKL185W | YLR106C |
| YOR167C | YOR058C | YOL151W | YOR066W |  | YKL186C | YLR121C |
| YOR180C | YOR062C | YOR009W | YOR083W |  | YKL208W | YLR178C |
| YOR185C | YOR066W | YOR019W | YOR100C |  | YKL208W | YLR183C |
| YOR229W | YOR083W | YOR058C | YOR167C |  | YKR009C | YLR238W |
| YOR287C | YOR100C | YOR062C | YOR180C |  | YKR009C | YLR249W |
| YOR291W | YOR167C | YOR066W | YOR185C |  | YKR009C | YLR254C |
| YPL066W | YOR180C | YOR083W | YOR229W |  | YKR027W | YLR263W |
| YPL090C | YOR185C | YOR100C | YOR287C |  | YKR097W | YLR292C |
| YPL104W | YOR229W | YOR167C | YOR291W |  | YLL021W | YLR305C |
| YPL112C | YOR287C | YOR180C | YPL066W |  | YLL040C | YLR312C |
| YPL133C | YOR291W | YOR185C | YPL090C |  | YLL046C | YLR409C |
| YPL189W | YPL066W | YOR229W | YPL104W |  | YLL056C | YLR447C |
| YPL200W | YPL090C | YOR287C | YPL112C |  | YLL056C | YML004C |
| YPL201C | YPL104W | YOR291W | YPL133C |  | YLR054C | YML031W |
| YPL206C | YPL112C | YPL066W | YPL189W |  | YLR054C | YML042W |
| YPL212C | YPL133C | YPL090C | YPL200W |  | YLR058C | YML059C |
| YPL252C | YPL163C | YPL104W | YPL201C |  | YLR058C | YML120C |
| YPL274W | YPL189W | YPL112C | YPL206C |  | YLR066W | YMR006C |
| YPR006C | YPL200W | YPL133C | YPL212C |  | YLR081W | YMR018W |
| YPR013C | YPL201C | YPL189W | YPL242C |  | YLR084C | YMR032W |
| YPR015C | YPL206C | YPL200W | YPL252C |  | YLR183C | YMR047C |
| YPR026W | YPL212C | YPL201C | YPL274W |  | YLR183C | YMR055C |
| YPR109W | YPL242C | YPL206C | YPR006C |  | YLR228C | YMR076C |
| YPR119W | YPL252C | YPL212C | YPR013C |  | YLR249W | YMR091C |
|  | YPL274W | YPL242C | YPR015C |  | YLR254C | YMR114C |
|  | YPR006C | YPL252C | YPR026W |  | YLR263W | YMR182C |
|  | YPR013C | YPL274W | YPR109W |  | YLR263W | YMR187C |
|  | YPR015C | YPR006C | YPR119W |  | YLR267W | YMR189W |
|  | YPR026W | YPR013C |  |  | YLR297W | YMR207C |
|  | YPR109W | YPR015C |  |  | YLR312C | YMR214W |
|  | YPR119W | YPR026W |  |  | YLR313C | YMR261C |
|  |  | YPR109W |  |  | YLR407W | YMR270C |
|  |  | YPR119W |  |  | YLR440C | YMR311C |
|  |  |  |  |  | YLR443W | YNL005C |
|  |  |  |  |  | YML004C | YNL014W |
|  |  |  |  |  | YML028W | YNL041C |
|  |  |  |  |  | YML042W | YNL058C |
|  |  |  |  |  | YML042W | YNL085W |
|  |  |  |  |  | YML059C | YNL108C |
|  |  |  |  |  | YMR006C | YNL152W |
|  |  |  |  |  | YMR018W | YNL176C |
|  |  |  |  |  | YMR018W | YNL196C |
|  |  |  |  |  | YMR032W | YNL208W |
|  |  |  |  |  | YMR047C | YNL234W |
|  |  |  |  |  | YMR055C | YNL270C |
|  |  |  |  |  | YMR076C | YNL306W |
|  |  |  |  |  | YMR100W | YNR015W |
|  |  |  |  |  | YMR153W | YNR044W |
|  |  |  |  |  | YMR188C | YOL007C |
|  |  |  |  |  | YMR189W | YOL017W |
|  |  |  |  |  | YMR189W | YOL034W |
|  |  |  |  |  | YMR280C | YOL066C |
|  |  |  |  |  | YMR290C | YOL084W |
|  |  |  |  |  | YNL014W | YOL091W |
|  |  |  |  |  | YNL014W | YOL104C |
|  |  |  |  |  | YNL024C | YOL151W |
|  |  |  |  |  | YNL041C | YOR006C |
|  |  |  |  |  | YNL053W | YOR009W |
|  |  |  |  |  | YNL058C | YOR019W |
|  |  |  |  |  | YNL117W | YOR058C |
|  |  |  |  |  | YNL175C | YOR066W |
|  |  |  |  |  | YNL192W | YOR083W |
|  |  |  |  |  | YNL196C | YOR099W |
|  |  |  |  |  | YNL196C | YOR100C |
|  |  |  |  |  | YNL208W | YOR180C |
|  |  |  |  |  | YNL234W | YOR185C |
|  |  |  |  |  | YNL234W | YOR259C |
|  |  |  |  |  | YNL261W | YOR287C |
|  |  |  |  |  | YNL265C | YOR301W |
|  |  |  |  |  | YNL270C | YPL007C |
|  |  |  |  |  | YNL306W | YPL066W |
|  |  |  |  |  | YNL306W | YPL090C |
|  |  |  |  |  | YNR002C | YPL100W |
|  |  |  |  |  | YNR002C | YPL104W |
|  |  |  |  |  | YNR015W | YPL112C |
|  |  |  |  |  | YNR015W | YPL121C |
|  |  |  |  |  | YOL007C | YPL183C |
|  |  |  |  |  | YOL017W | YPL200W |
|  |  |  |  |  | YOL017W | YPL201C |
|  |  |  |  |  | YOL038W | YPL203W |
|  |  |  |  |  | YOL066C | YPL235W |
|  |  |  |  |  | YOL084W | YPL252C |
|  |  |  |  |  | YOL088C | YPR006C |
|  |  |  |  |  | YOL091W | YPR015C |
|  |  |  |  |  | YOL091W | YPR026W |
|  |  |  |  |  | YOL104C | YPR119W |
|  |  |  |  |  | YOL104C | YPR125W |
|  |  |  |  |  | YOL154W |  |
|  |  |  |  |  | YOR009W |  |
|  |  |  |  |  | YOR009W |  |
|  |  |  |  |  | YOR016C |  |
|  |  |  |  |  | YOR019W |  |
|  |  |  |  |  | YOR058C |  |
|  |  |  |  |  | YOR058C |  |
|  |  |  |  |  | YOR066W |  |
|  |  |  |  |  | YOR077W |  |
|  |  |  |  |  | YOR083W |  |
|  |  |  |  |  | YOR100C |  |
|  |  |  |  |  | YOR100C |  |
|  |  |  |  |  | YOR100C |  |
|  |  |  |  |  | YOR167C |  |
|  |  |  |  |  | YOR180C |  |
|  |  |  |  |  | YOR185C |  |
|  |  |  |  |  | YOR185C |  |
|  |  |  |  |  | YOR210W |  |
|  |  |  |  |  | YOR260W |  |
|  |  |  |  |  | YOR287C |  |
|  |  |  |  |  | YOR291W |  |
|  |  |  |  |  | YOR316C |  |
|  |  |  |  |  | YOR384W |  |
|  |  |  |  |  | YPL006W |  |
|  |  |  |  |  | YPL065W |  |
|  |  |  |  |  | YPL066W |  |
|  |  |  |  |  | YPL090C |  |
|  |  |  |  |  | YPL104W |  |
|  |  |  |  |  | YPL104W |  |
|  |  |  |  |  | YPL112C |  |
|  |  |  |  |  | YPL112C |  |
|  |  |  |  |  | YPL112C |  |
|  |  |  |  |  | YPL171C |  |
|  |  |  |  |  | YPL179W |  |
|  |  |  |  |  | YPL200W |  |
|  |  |  |  |  | YPL200W |  |
|  |  |  |  |  | YPL201C |  |
|  |  |  |  |  | YPL201C |  |
|  |  |  |  |  | YPL212C |  |
|  |  |  |  |  | YPL252C |  |
|  |  |  |  |  | YPL274W |  |
|  |  |  |  |  | YPR006C |  |
|  |  |  |  |  | YPR013C |  |
|  |  |  |  |  | YPR015C |  |
|  |  |  |  |  | YPR022C |  |
|  |  |  |  |  | YPR026W |  |
|  |  |  |  |  | YPR089W |  |
|  |  |  |  |  | YPR109W |  |
|  |  |  |  |  | YPR109W |  |
|  |  |  |  |  | YPR119W |  |

**Table S3: List of yeast strains**

| **Strain** | **Genotype** | **Source** | **Method** |
| --- | --- | --- | --- |
| yFMP007 | *MATa his3Δ1 leu2Δ0 met15Δ0 ura3Δ0 isw2Δ::KANMX4* | Euroscarf | - |
| yFMP009 | *MATα his3Δ1 leu2Δ0 lys2Δ0 ura3Δ0* | Euroscarf | - |
| yFMP010 | *MATa his3Δ1 leu2Δ0 met15Δ0 ura3Δ0 itc1Δ::kanMX4* | Euroscarf | - |
| yFMP012 | *MATa his3Δ1 leu2Δ0 met15Δ0 ura3Δ0* | Euroscarf | - |
| yFMP039 | *MATa his3Δ1 leu2Δ0 met15Δ0 ura3Δ0 itc1::itc1∆2-374-HIS3* colony 1 | - | Transformation in yFMP012 |
| yFMP040 | *MATa his3Δ1 leu2Δ0 met15Δ0 ura3Δ0 itc1::itc1∆2-374-HIS3* colony 2 | - | Transformation in yFMP012 |
| yFMP041 | *MATa his3Δ1 leu2Δ0 met15Δ0 ura3Δ0 itc1::itc1∆2-374-HIS3* colony 3 | - | Transformation in yFMP009 |
| yFMP168 | *MATa his3Δ1 leu2Δ0 met15Δ0 ura3Δ0 isw2::isw2-TAP:LEU2* | - | Transformation in yFMP706 |
| yFMP197 | *MATa his3Δ1 leu2Δ0 met15Δ0 ura3Δ0 isw2::isw2-TAP:LEU2 itc1::itc1-FLAG:HIS3* colony 1 | - | Transformation in yFMP168 |
| yFMP198 | *MATa his3Δ1 leu2Δ0 met15Δ0 ura3Δ0 isw2::isw2-TAP:LEU2 itc1::itc1-FLAG:HIS3* colony 2 | - | Transformation in yFMP168 |
| yFMP200 | *MATa his3Δ1 leu2Δ0 met15Δ0 ura3Δ0 isw2::isw2-TAP:LEU2 itc1::itc1∆2-374:FLAG:HIS3* colony 1 | - | Transformation in yFMP168 |
| yFMP202 | *MATa his3Δ1 leu2Δ0 met15Δ0 ura3Δ0 isw2::isw2-TAP:LEU2 itc1::itc1∆2-374:FLAG:HIS3* colony 2 | - | Transformation in yFMP168 |
| yFMP298 | *MATa/MATα his3Δ1/his3Δ1 leu2Δ0/leu2Δ0*  *LYS2/lys2Δ0 met15Δ0/MET15 ura3Δ0/ura3Δ0 ITC1/itc1∆2-374 colony 1* | - | Transformation in yFMP721 |
| yFMP299 | *MATa/MATα his3Δ1/his3Δ1 leu2Δ0/leu2Δ0*  *LYS2/lys2Δ0 met15Δ0/MET15 ura3Δ0/ura3Δ0 ITC1/itc1∆2-374 colony 2* | - | Transformation in yFMP721 |
| yFMP706 | *MATa his3Δ1 leu2Δ0 met15Δ0 ura3Δ0 isw2::isw2-TAP::HIS3MX6* | Open Biosystems | - |
| yFMP718 | *MATa his3Δ1 leu2Δ0 met15Δ0 ura3Δ0 itc1::itc1∆2-374-HIS3 isw2::LEU2* | - | Transformation in yFMP039 |
| yFMP721 | *MATa/MATα his3Δ1/his3Δ1 leu2Δ0/leu2Δ0 lys2Δ0/LYS2 MET15/met15Δ0 ura3Δ0/ura3Δ0* | - | Mating yFMP009 and yFMP012 |
| yFMP296 | *MATα his3Δ1 leu2Δ0 lys2Δ0 ura3Δ0 chd1::HYG* | - | Transformation in yFMP009 |
| yFMP001 | *MATa his3Δ1 leu2Δ0 met15Δ0 ura3Δ0 isw1::kanMX* | Euroscarf | - |
| yFMP294 | *MATa/MATα his3Δ1/his3Δ1 leu2Δ0/leu2Δ0 lys2Δ0/LYS2 MET15/met15Δ0 ura3Δ0/ura3Δ0 ISW1/isw1::kanMX CHD1/chd1::HYG* | - | Mating yFMP296 and yFMP001 |
| yFMP360 | *MATa his3Δ1 leu2Δ0 met15Δ0 ura3Δ0 isw1::kanMX chd1::HYG* | - | Sporulation and tetrad dissection yFMP294 |
| yFMP362 | *MATα his3Δ1 leu2Δ0 met15Δ0 ura3Δ0 isw1::kanMX chd1::HYG* | - | Sporulation and tetrad dissection yFMP294 |
| yFMP373 | *MATa/MATα his3Δ1/his3Δ1 leu2Δ0/leu2Δ0 lys2Δ0/LYS2 MET15/met15Δ0 ura3Δ0/ura3Δ0 ISW1/isw1::kanMX CHD1/chd1::HYG ISW2/isw2::kanMX* | - | Mating yFMP362 and yFMP007 |
| yFMP377 | *MATa his3Δ1 leu2Δ0 met15Δ0 ura3Δ0 isw1::kanMX chd1::HYG isw2::kanMX* | - | Sporulation and tetrad dissection yFMP373 |
| yFMP378 | *MATα his3Δ1 leu2Δ0 met15Δ0 ura3Δ0 isw1::kanMX chd1::HYG isw2::kanMX* | - | Sporulation and tetrad dissection yFMP373 |
| yFMP428 | *MATa/MATα his3Δ1/his3Δ1 leu2Δ0/leu2Δ0 lys2Δ0/LYS2 MET15/met15Δ0 ura3Δ0/ura3Δ0 ISW1/isw1::kanMX CHD1/chd1::HYG ITC1/ itc1∆2-374-HIS3* |  | Mating yFMP362 and yFMP039 |
| yFMP400 | *MATa his3Δ1 leu2Δ0 met15Δ0 ura3Δ0 isw1::kanMX chd1::HYG itc1∆2-374-HIS3* | - | Sporulation and tetrad dissection yFMP428 |
| yFMP401 | *MATα his3Δ1 leu2Δ0 met15Δ0 ura3Δ0 isw1::kanMX chd1::HYG itc1∆2-374-HIS3* | - | Sporulation and tetrad dissection yFMP428 |

**Table S4: List of oligonucleotides**

| **Oligo No.** | **Sequence** | **Comments** | **HPLC purified** |
| --- | --- | --- | --- |
| oFMP456 | GGATAATACCGATCAGAAAAATCC | Itc1 locus check F | No |
| oFMP457 | CAGAAACTGATAGGCTTGTAATAGC | Itc1 locus check R | No |
| oFMP502 | GTTTGATATCGATATTTCAGAAGCTG | Itc1 locus check R | No |
| oFMP513 | GAAACCTATATTACTTCCTGATC | Itc1 seq 1 | No |
| oFMP514 | CACTTTCACTAAGCACTTAATC | Itc1 seq 2 | No |
| oFMP515 | GTCTCTCTCATTTTAGCCTTG | Itc1 seq 3 | No |
| oFMP516 | GAACTACCGCAATGTTAACTG | Itc1 seq 4 | No |
| oFMP517 | CTACAAAGATTAAAGCCGTTG | Itc1 seq 5 | No |
| oFMP518 | CACCAAAAATGAAGAAAAGTTG | Itc1 seq 6 | No |
| oFMP572 | TTCTCATTGAAGTTTTTACTGC | Itc1 seq 3.1 | No |
| oFMP573 | CATGGAACTATGTGGTCAA | Itc1 seq 4.1 | No |
| oFMP574 | GTTCCGGATCTTATAATTACG | Itc1 seq 5.1 | No |
| oFMP575 | TCCATAATAAATTCGCAGAG | Itc1 seq 6.1 | No |
| oFMP700 | ATTTGGGGCAGGTGTTAC | Itc1 FLAG C-terminus F | No |
| oFMP701 | TAAGCTTGGTGAGCGCTAG | Itc1 FLAG C-terminus R | No |
| oFMP702 | CAGTAACACCTGCCCCAAATGACTATAAGGACCACGACG | FLAG F | No |
| oFMP703 | CCTAGCGCTCACCAAGCTTACTTATCGTCATCGTCTTTG | FLAG R | No |
| oFMP722 | CTGAGGCTCCATGAATTCGATATCAAGCTTATCG | Delete Itc1 N-terminus F | No |
| oFMP723 | GAATTCATGGAGCCTCAGGCTGTAACAATAAC | Delete Itc1 N-terminus R | No |
| oFMP773 | AAGAAAATAACAATAGGAGGAAGTAAAGAAAGCCGTTAATAAACAATGGAGCCTCAGGCTGTAACA | Transform Itc1 N-terminus F | Yes |
| oFMP544 | GAATACTACAATTTACCATCAGTTACAAAGGAAGTTTTTTATATACTACATAAGAACACCTTTGGTGG | Transform Itc1 N-terminus R | Yes |
| oFMP628 | CTTATATCAATGGTAGCTGCTTC | ISW1 locus check F | No |
| oFMP629 | GCAGTATAAAGAATTGGAAGAAC | ISW1 locus check R | No |
| oFMP671 | CATGGTATTTTGCGCTACTCG | ISW2 locus check F | No |
| oFMP672 | GCAGGCTTTAGTTTTCAGCA | ISW2 locus check R | No |
| oFMP673 | TCCGGACATCTAAGTCAAGTTG | CHD1 locus check F | No |
| oFMP674 | GAACTGGAGCGAAAGAGAAC | CHD1 locus check R | No |

**Fig. S1. Sequence and structure of the WAC-downWAC module. (A)** Sequence homology of the downWAC region. (**B)** WAC and downWAC jointly form a positively charged interface. Electrostatic surface potentials of the WAC (left; downWAC is yellow) and downWAC region (right; WAC is orange).

**Fig. S2. Representative tetrad dissection results from an independent experiment.**

**Fig. S3.** Spot dilution assay of cells lacking Itc1 or with different variants of Itc1 with or without the FLAG tag. Ten-fold dilutions are shown.

**Fig. S4.** Uncropped blot (related to Fig. 3C).

**Fig. S5. Individual composite plots of indicated yeast mutants overlapped with WT sample for all samples shown in Fig. 4A. (A)** Composite plot for all genes. **(B)** Same as A but for Isw2-dependent genes.

**Fig. S6. Composite plots of WT and indicated yeast mutants in W303 cells from Donovan et al study. (A)** Composite plot for all genes. **(B)** Same as A but for Isw2-dependent genes. The *itc1*^ΔWAC^ refers to deletion of amino acids 24-130.

**Fig. S7.** **Deletion of the N-terminus of Itc1 does not affect nucleosome organization in *MATα*** ***isw1Δ chd1Δ* sensitive background.** Composite plots showing nucleosome organization of all yeast genes (left) and genes exhibiting a shift in the +1-nucleosome location by at least 10 bp in *isw2Δ* strain (right) in the indicated yeast strains in *MATα* (alpha) background.

**Fig. S8. Individual composite plots of indicated yeast mutants overlapped with WT sample for all samples shown in Fig. 6B. (A)** Composite plot for all genes. **(B)** Same as A but for Isw2-dependent genes.
